# Interesting to All

**Published:** 1876-01

**Authors:** 


### INTERESTING TO ALL.

Everybody uses soap, or ought to, and so everybody is interested in the soap question. We do not think all mankind are as fully impressed with the importance of purity in this substance as they ought to be; if we did we should not consider it necessary to hammer away at them from month to month regarding the difference between soap made from gross, unwholesome, decomposed animal grease, and soap manufactured from pure, aromatic, and wholesome vegetable oils. Now and then a case occurs in the practice of every physician, in which serious injury can be directly traced to bad soap. One is now reported by a professional gentleman here. A gentleman had a small boil on his hand, and applied to it a plaster of soap and sugar. In a few hours it became evident that he was poisoned; and, in spite of all that could be done to save him, he died in 36 hours. The decision of his medical attendants was, that the soap was made from animal fats, rendered poisonous from decay, and that the innoculation with this terrible virus, killed him. The unbroken healthy skin may be washed with such diseased and poisonous stuff with a possible impunity; but if the skin is wounded, or a pimple exists, the contact of disease is highly dangerous. The tender skin of the new-born babe is washed with soap which seems nice because sweetly scented, but it carries with it a multitude of fearful ills in the way of skin-diseases and eruptions, often lasting through life. Let us look closely to our soaps. Let us be as sure as we can be of anything, that we are making use of soaps made only from vegetable substances and not from the decaying carcasses of horses, and the old bones in which the "soap-fat man" delights.

As we do not like to mention an evil without indicating a remedy, let us remark that Packer's Tar Soap is a genuine vegetable soap, valuable as a detergent or cleanser, and possessing some specific virtues as a neutralizing agent in diseased conditions. It is a very soft, pleasant, emollient soap to use upon the skin; and, when followed by a few drops of "Packer's Charm,"—a soothing and healing preparation of real merit,—nothing further is needed in the direction of beautifiers, soothers, and healers. To those whose occupation is in any degree offensive, we can especially commend these delicate preparations.

---

### PACKER'S TAR SOAP AND CHARM.

We have prevailed upon our neighbors to keep a supply of these elegant articles constantly on hand. So if our readers do not find them at the druggist's, let them apply to the Health Food Company.

